# Sol-Gel Synthesis of Carbon Xerogel-ZnO Composite for Detection of Catechol

**DOI:** 10.3390/ma9040282

**Published:** 2016-04-12

**Authors:** Dawei Li, Jun Zang, Jin Zhang, Kelong Ao, Qingqing Wang, Quanfeng Dong, Qufu Wei

**Affiliations:** 1 Key Laboratory of Eco-Textiles, Ministry of Education, Jiangnan University, Wuxi 214122, China; 7130707015@vip.jiangnan.edu.cn (D.L.); 6140708013@vip.jiangnan.edu.cn (J.Z.); 1090112401@vip.jiangnan.edu.cn (K.A.); qqwang@jiangnan.edu.cn (Q.W.); 2 State Key Laboratory for Physical Chemistry of Solid Surfaces, Collaborative Innovation Center of Chemistry for Energy Materials, and Department of Chemistry, College of Chemistry and Chemical Engineering, Xiamen University, Xiamen 361005, China; jzang@ncsu.edu (J.Z.); qfdong@xmu.edu.cn (Q.D.)

**Keywords:** carbon xerogel, ZnO, laccase, phenolic biosensor

## Abstract

Carbon xerogel-zinc oxide (CXZnO) composites were synthesized by a simple method of sol-gel condensation polymerization of formaldehyde and resorcinol solution containing zinc salt followed by drying and thermal treatment. ZnO nanoparticles were observed to be evenly dispersed on the surfaces of the carbon xerogel microspheres. The as-prepared CXZnO composites were mixed with laccase (Lac) and Nafion to obtain a mixture solution, which was further modified on an electrode surface to construct a novel biosensing platform. Finally, the prepared electrochemical biosensor was employed to detect the environmental pollutant, catechol. The analysis result was satisfactory, the sensor showed excellent electrocatalysis towards catechol with high sensitivity (31.2 µA·mM^−1^), a low detection limit (2.17 µM), and a wide linear range (6.91–453 µM). Moreover, the biosensor also displayed favorable repeatability, reproducibility, selectivity, and stability besides being successfully used in the trace detection of catechol existing in lake water environments.

## 1. Introduction

A biosensor, as a type of analytical tool, can be employed to detect analyte existing in various environments. It consists of a biological recognition component and a physicochemical transduction device [1]. Since Leland C. Clark invented enzyme based electrodes in 1962, enzyme based biosensors have attracted a great deal of attention from scientists and researchers [2,3,4]. Due to the great advantages of enzyme biosensors over conventional analytical techniques, such as low price, high sensitive/selective, rapid response, and amenable miniaturization, *etc.*, they have been applied in multifarious fields, such as clinical medicine, environment monitoring, and food safety, as well as homeland security [5,6]. Laccase (Lac) is a multicopper oxidase which can catalyze phenolic compounds to give their oxidation form accompanied by reduction of molecular oxygen [7]. As a consequence, numerous Lac based biosensors have been prepared to detect phenols in tea infusions and wines, as well as in watery environments [8,9,10].

In order to improve the sensitivity and selectivity of biosensors, a variety of conductive materials or conductive nanomaterials have been added to the biosensing system, which mainly contain metal nanoparticles, metal oxide nanoparticles, carbon materials, and conductive polymers. Among these materials, carbon materials, including carbon black, carbon nanotube, graphene, carbon nanofiber, mesoporous carbon, *etc.*, are commonly used due to their low cost, outstanding electron transfer ability, good chemical stability, and biocompatibility [11]. Specifically as a type of porous carbon material, carbon xerogel (CX) harbors many merits, e.g., low mass density, large surface area, and excellent electrical conductivity. On the basis of these advantages, CX has been widely applied in adsorbents, catalyst supports, and electrode materials for supercapacitors and rechargeable lithium-ion batteries. Only a few literature examples have reported biosensors utilizing CX which possesses a huge application potential in biosensors. Zinc oxide (ZnO), as an admirable semiconductor material, has attracted wide attention in various application fields, such as piezoelectric devices, sensors, transparent electronics, optics, optoelectronics, and actuators [12,13]. The satisfactory electron conduction ability, good biocompatibility, and chemical stability renders ZnO to be an outstanding modification material in biosensors [14]. Besides, the isoelectric point (IEP) of ZnO is about 9.5, which is very favorable for adsorption of proteins with low IEP [15]. However, direct modification of ZnO nanoparticles on electrodes usually leads to their aggregation, which considerably restricts their electrocatalysis and electrochemical performance. Combination of ZnO nanoparticles with conductive substrate materials can effectively solve this problem.

In this work, we synthesized carbon xerogel-zinc oxide (CXZnO) composites through a simple method of sol-gel condensation polymerization of formaldehyde and resorcinol solution containing zinc salt followed by drying and thermal treatment. ZnO nanoparticles were evenly dispersed on the surfaces of the carbon xerogel microspheres in the final products. The as-prepared CXZnO composites were further employed to modify the electrode with Lac and Nafion to construct a novel biosensing platform. The obtained biosensor showed excellent bio-electrocatalysis towards the phenolic compound catechol, with high sensitivity, low detection limit and a wide linear range. Moreover, the sensor demonstrated its practical application potential by detecting catechol existing in real lake water with satisfactory recovery. Our study expands the application of carbon xerogel in the biosensing field and offers theoretical support for exploiting high-efficient enzyme based biosensors.

## 2. Materials and Methods

### 2.1. Chemicals and Reagents

Laccase (Lac, enzyme activity ≥10 U/mg) from Trametes versicolor was purchased from Sigma-Aldrich. Nafion (5% w/w) was obtained from Shanghai Branch, Du Pont China Holding Co., Ltd. (Shanghai, China). Catechol was purchased from Shanghai Aladdin Chemical Reagent Company (Shanghai, China). Zinc acetate dihydrate (C_4_H_6_O_4_Zn·2H_2_O), formaldehyde, resorcinol, and other chemicals were purchased from the Sinopharm Group Chemical Reagent Co., Ltd. (Shanghai, China). All of the chemicals were of analytical grade and used without further purification. In addition, acetate buffer solution (0.1 M HAc–NaAc, pH = 5.0) was used as a supporting electrolyte. All aqueous solutions were prepared with deionized water (DIW).

### 2.2. Apparatus

TGA measurement was conducted using a Mettler Toledo analyzer in an air atmosphere, the temperature range was from ambient temperature to 800 °C with a heating rate of 10 °C/min. The chemical components of CXZnO composites were analyzed by a Powder D8 Advance X-ray diffraction (XRD, Bruker AXS D8, Coventry, UK). The nitrogen absorption and desorption isotherms of CXZnO composites at 77 K were measured by using a TriStar 3020 surface area and pore analyzer (Micromeritics, America). The morphologies of CXZnO composites were observed by using a field emission scanning electron microscope (FE-SEM, Hitachi S-4800, Tokyo, Japan) and a high-resolution transmission electron microscope (TEM, JEOL/JEM-2100, Tokyo, Japan). Prior to scanning under the FE-SEM, the samples were sputter coated with gold for 90 s to avoid charge accumulations. Electrochemical experiments were conducted at room temperature using a CHI 660E electrochemical workstation (CH Instruments, Inc., Shanghai, China). A three-electrode cell with a glass carbon electrode (GCE) (3.0 mm in diameter, purchased from Gaoss Union Technology Co., Ltd., Wuhan, China), a platinum wire auxiliary electrode, and an Ag/AgCl reference electrode were used for electrochemical measurements. The electrolyte solution was bubbled with highly pure nitrogen for 15 min before electrochemical experiments and a nitrogen atmosphere was kept over the solution throughout the experiments except for the amperometric experiments.

### 2.3. Synthesis of CXZnO Composites

The synthesis procedure of CXZnO composites can be described as follows: 1.375 g of resorcinol and 2.23 g of C_4_H_6_O_4_Zn·2H_2_O were dissolved in 10 mL of DIW with the aid of stirring and ultrasonication followed by adding 2.375 mL of formaldehyde into the formed solution. After fast stirring for a while, the solution was sealed by plastic wrap and placed in an oven at 76 °C. Through a sol-gel reaction for three days, the plastic wrap was poked to dry the composite gel. Eventually, the dried gel was put into a high temperature tube furnace to carbonize it in a N_2_ atmosphere. The heating rate was 5 °C/min, maintaining 300 °C and 800 °C for 1 h and 2 h, respectively, and cooling down to room temperature. The final products were solely CXZnO composites, and the composites were further ground to powders for the following experiments. For comparison, ZnO powders were prepared by directly heating C_4_H_6_O_4_Zn·2H_2_O with the same thermal treatment process.

### 2.4. Preparation of Biosensors

The preparation procedures of biosensors are described as follows: 1.5 mg of CXZnO was added into actetate buffer of pH = 5.0 and with the aid of ultrasonication stirring, a CXZnO suspension was obtain. Subsequently, 15 mg of Lac and 75 µL of Nafion solution (5 wt %) were added to the above CXZnO suspension, by which the final mixture solution was achieved. Eventually, 10 µL of the mixture was dropped onto a freshly polished glass carbon electrode (GCE) surface to fabricate the biosensor, and the dried modified GCE was named GCE/Lac-CXZnO-Nafion, which was stored at 4 °C for use.

GCE/Lac-Nafion and GCE/Lac-ZnO-Nafion modified electrodes were fabricated by a similar methods with the same amount of Lac for comparison experiments. All the modified electrodes were immersed into a buffer for 30 min to remove impurities before electrochemical measurements.

## 3. Results and Discussion

### 3.1. Characterization of CXZnO Composites

**Figure 1:**
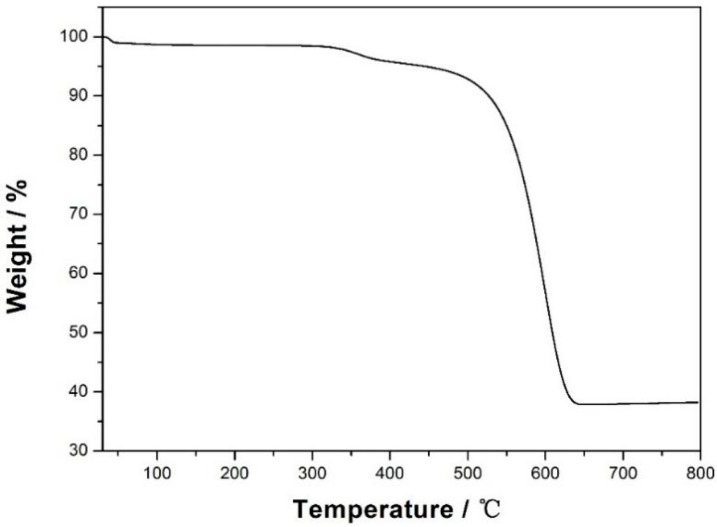
Thermogravimetric analysis (TGA) curve of CXZnO composites.

Thermogravimetric analysis (TGA) characterization was employed to determine the contents of carbon xerogel in CXZnO composites. The result is displayed in Figure 1, which shows the weight loss of the CXZnO composites terminated at around 640 °C. The weight loss below 640 °C can be ascribed solely to carbon, hence, the content of ZnO in the composites can be assessed in Figure 1, which is about 40%.

**Figure 2:**
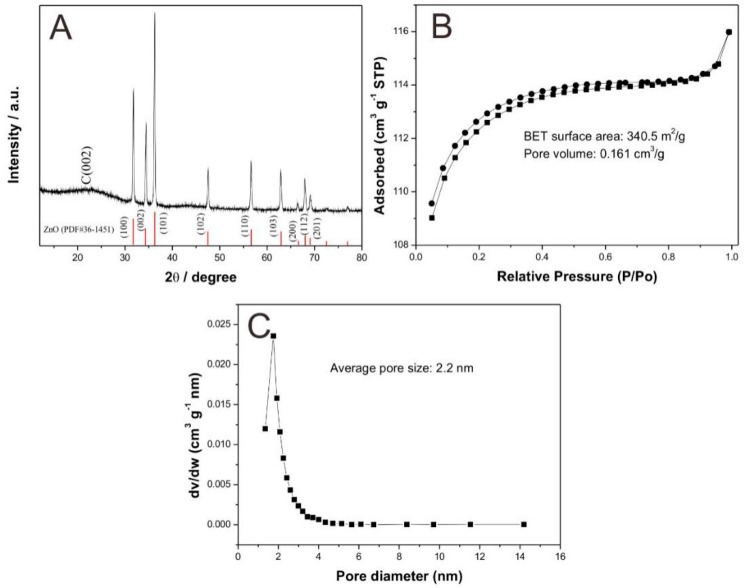
X-ray diffraction (XRD) pattern (**A**); nitrogen adsorption-desorption isotherm (**B**); and pore size distribution curve (**C**) of CXZnO composites.

X-ray diffraction (XRD) analysis was used to investigate the chemical ingredients and crystal structures of the CXZnO composites, and the result is shown in Figure 2A. The diffraction peak occurring at about 24° is attributed to the {0 0 2} plane of graphite carbon [16]. In addition, the peaks at *ca.* 31.8°, 34.4°, 36.3°, 47.5°, 56.6°, 62.9°, 66.3°, 67.9°, and 69.1° correspond to the {1 0 0}, {0 0 2}, {1 0 1}, {1 0 2}, {1 1 0}, {1 0 3}, {2 0 0}, {1 1 2} and {2 0 1} crystalline planes of zinc oxide, respectively [17]. The XRD result demonstrates that the CXZnO composite was successfully synthesized.

Figure 2B displays the nitrogen adsorption-desorption isotherm of CXZnO composites. According to the BDDT classification [18], the isotherm belongs to a combination of type I and type II of micro-mesoporous materials. Meanwhile, the specific surface area of CXZnO composites was tested to be 340.5 m^2^/g with a pore volume of 0.161 cm^3^/g, and the average pore size was about 2.2 nm through the Barrett-Joyner-Halenda (BJH) model (Figure 2C). The large specific surface area and special micro-mesoporous structure were both favorable for the immobilization of biological enzyme protein, leading to high bioelectrocatalytic properties.

**Figure 3:**
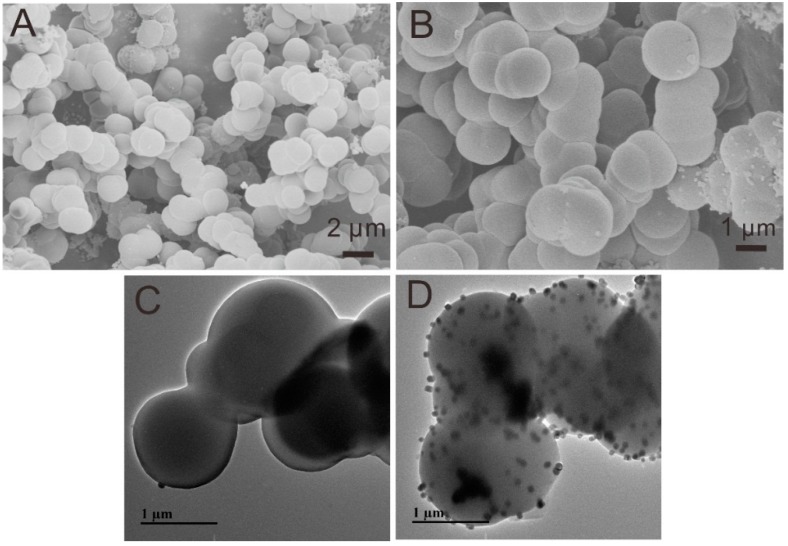
Scanning electon microscopy (SEM) topographies of CXZnO composites (**A**); partial enlarged drawing of SEM image of CXZnO composites (**B**); transmission electron microscopy (TEM) images of carbon xerogel (**C**) and CXZnO composites (**D**).

Figure 3A shows the SEM morphology image of CXZnO composites. A large number of microspheres were gathered together, some of which formed a chain-like structure. The average diameter of these microspheres was about 1.8 µm. It can be seen from the enlarged SEM image of CXZnO composites (Figure 3B) that most microspheres possessed smooth surfaces while there were also some nanoparticle loaded microspheres, which may be the CXZnO composite microspheres. Figure 3C,D displays the TEM image of CXZnO composites, it can be clearly observed that there are two kinds of morphologies, one shows smooth surfaces with no nanoparticles, and the other has rough surfaces with evenly distributed nanoparticles. The average diameter of these nanoparticles is around 103 nm. So the small sizes and uniform distribution of the ZnO nanoparticles were both favorable to perform their electrocatalytic activities. Meanwhile, the nanoparticles also offered abundant active sites for Lac immobilization.

### 3.2. Electrochemical Studies of Modified Electrodes

**Figure 4:**
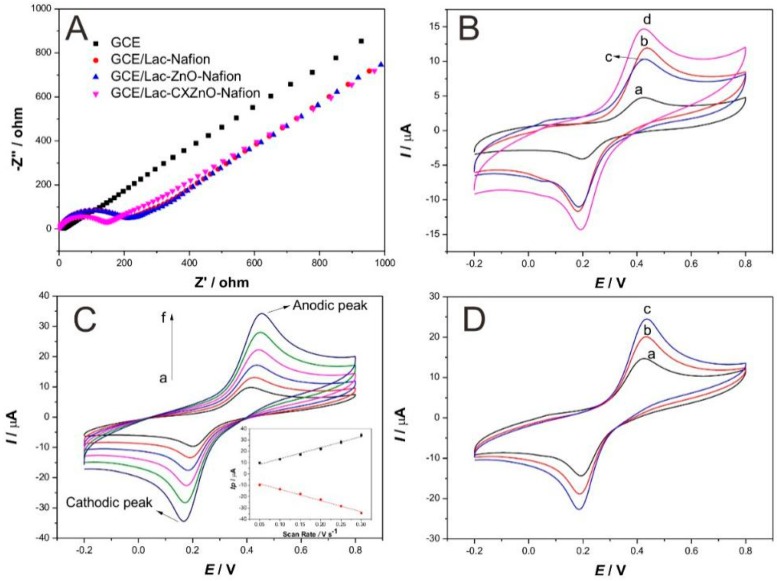
(**A**) Electrochemical impedance spectroscopy (EIS) of modified electrodes: bare GCE, GCE/Lac-Nafion, GCE/Lac-ZnO-Nafion, and GCE/Lac-CXZnO-Nafion in 0.1 M KCl containing 5 mM Fe(CN)_6_^3−/4−^. Frequency range: 0.01 Hz–100,000 Hz. Amplitude: 5 mV; (**B**) Cyclic voltammograms of (a) bare GCE; (b) GCE/Lac-Nafion; (c) GCE/Lac-ZnO-Nafion; and (d) GCE/Lac-CXZnO-Nafion in pH 5.0 acetate buffer solution containing 100 µM catechol at 100 mV/s; (**C**) Cyclic voltammograms of GCE/Lac-CXZnO-Nafion in pH = 5.0 acetate buffer solution containing 100 µM catechol at scan rates of 50, 100, 120, 150, 200, 250, and 300 mV/s (a–f), respectively. Inset: Plots of the corresponding anodic and cathodic peak currents *vs.* scan rate; (**D**) Electrocatalysis of GCE/Lac-CXZnO-Nafion towards catechol in pH = 5.0 acetate buffer solution with scan rate 100 mV/s. Catechol concentrations (µM): (a) 100; (b) 200; (c) 300.

Electrochemical Impedance Spectroscopy (EIS) characterization was utilized to compare the interface resistances of different modified electrodes, the result of the Nyquist plot of impedance is shown in Figure 4A. The values of charge transfer resistance (Rct) of modified electrodes are proportional to the diameters of semicircles. Apparently, bare electrode (GCE) shows almost a straight line, indicating the negligible Rct value. However, an obvious semicircle occurred on the curve for GCE/Lac-Nafion, the Rct value is about 227 Ω, suggesting that the interface resistance was increased by the immobilization process of Lac. In addition, the Rct value of GCE/Lac-ZnO-Nafion was almost equal to that of GCE/Lac-Nafion, implying that the addition of ZnO did not decrease the interface resistance of the modified electrode. This may be explained by the fact that ZnO is a type of semiconductor material, which may possess similar electron conductive ability to Lac. While, the semicircle diameter of the curve for GCE/Lac-CXZnO-Nafion was smaller than those of GCE/Lac-Nafion and GCE/Lac-ZnO-Nafion, the Rct value was also decreased to 145 Ω. This demonstrated that the CXZnO composites accelerated the electron transfer thereby weakening the interface resistance of the modified electrode hence revealing the excellent electron conductivity of carbon xerogel.

**Scheme 1:**
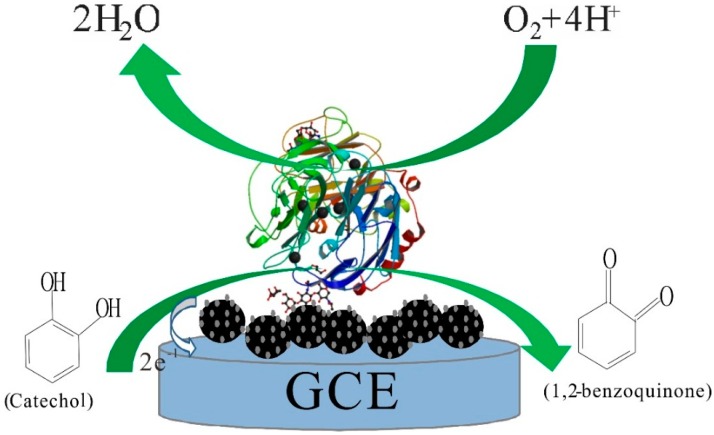
Schematic illustration of the catalyzed oxidation of catechol by laccase on the electrode surface.

The electrocatalytic properties of four modified electrodes, including bare GCE, GCE/Lac-Nafion, GCE/Lac-ZnO-Nafion, and GCE/Lac-CXZnO-Nafion, were compared using the cyclic voltammograms of these electrodes in pH = 5.0 acetate buffer solution containing 100 µM catechol, the result is shown in Figure 4B. These electrodes all show a pair of distinct redox peaks, which can be ascribed to the redox electrochemical reaction of catechol occurring on the electrode surface. It was observed that bare GCE possessed the smallest oxidation and reduction peak current values, indicating the poor electrocatalytic activity of bare GCE. GCE/Lac-Nafion presented higher peak current values as compared to bare GCE, the oxidation peak current value reached to 12 µA, and the reduction peak current value was *ca.* 11.67 µA. This can be attributed to the high-efficient catalysis of Lac toward catechol. The peak current values for GCE/Lac-ZnO-Nafion were decreased to some extent, which suggested that the aggregated ZnO particles may impair the electrocatalytic activity of the modified electrode. It is noticeable that GCE/Lac-CXZnO-Nafion shows the largest redox peak current values, which were increased to 14.68 µA and 14.31 µA, respectively. This demonstrated that the CXZnO composites can enhance the electrocatalytic properties of the modified electrode, maybe attributing to the good conductivity of CXZnO composites and the synergetic catalysis of ZnO nanoparticles. The whole electrochemical reaction was a quasi-reversible cyclic process and the sensing mechanism of Lac on GCE/Lac-CXZnO-Nafion is illustrated in Scheme 1. Under the presence of molecular oxygen, the catechol was oxidized to 1,2-benzoquinone by Lac, coupled with the electrocatalytic reduction of oxygen to water on the surface of GCE. The reaction process can be described as follows:

Catechol + Lac(oxy) → 1,2-Benzoquinone + Lac(deoxy) + 2H^+^ + 2e^−^ Lac(deoxy) + O_2_ + 4H^+^ → Lac(oxy) + 2H_2_O (1)

Figure 4C shows the influence of scan rates on the cyclic voltammograms of GCE/Lac-CXZnO-Nafion. As the scan rates grew from 50 mV/s to 300 mV/s, both the anodic peak and cathodic peak current values increased. It can be seen from the inset of Figure 4C, the peak current values enhanced linearly with the scan rates and were proportional to the scan rates. This indicated that the electrochemical conduction occurring on the electrode surface was a surface-controlled electrochemical reaction process. Figure 4D displays the electrocatalysis of GCE/Lac-CXZnO-Nafion towards catechol with different concentrations. Obviously, the current values of redox peaks increased with the increment of substrate (catechol) concentration, implying that GCE/Lac-CXZnO-Nafion possessed exceptionally good electrocatalytic properties toward catechol and can be applied in a catechol enzyme-based biosensor.

### 3.3. Analytical Performance for Detecting Catechol

**Figure 5:**
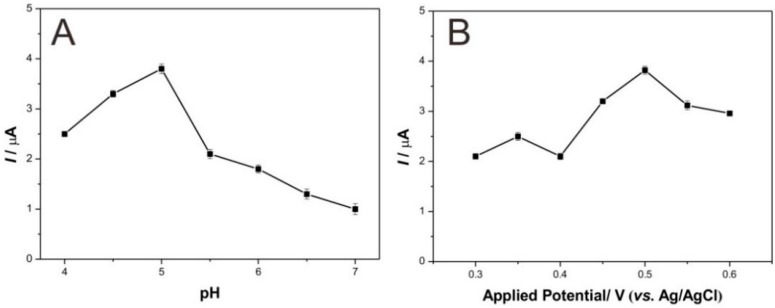
(**A**) The pH effects; and (**B**) applied potentials of GCE/Lac-CXZnO-Nafion on the catalytic currents of catechol in 0.1 M acetate buffer solution containing 100 µM catechol.

Chrono-amperometry was employed to investigate the analytical performance of the as-prepared biosensor for detecting catechol. To acquire the optimal current response and the highest sensitivity of the biosensor, before the amperometric tests, some parameters like solution pH and applied work voltage were optimized. As shown in Figure 5, the optimal pH and applied potential were pH 5.0 and 0.5 V, respectively, which were fixed in the following chrono-amperometry tests.

**Figure 6:**
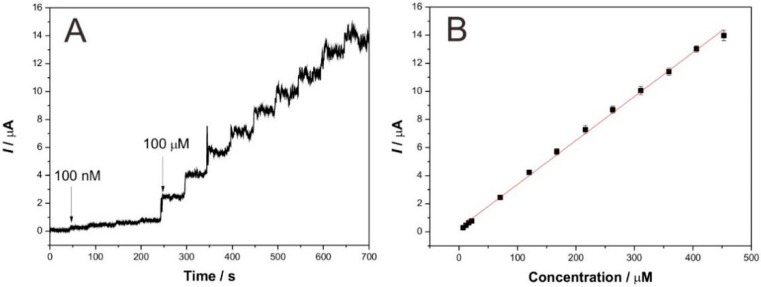
(**A**) Typical current-time response curve of the GCE/Lac-CXZnO-Nafion upon successive additions of catechol into pH = 5.0 acetate buffer solution, applied potential: 0.5 V; (**B**) Calibration curve of steady-state currents *vs.* catechol concentrations.

Figure 6A depicts the typical current-time response curve of the GCE/Lac-CXZnO-Nafion upon successive additions of catechol into pH = 5.0 acetate buffer solution with 0.5 V of applied potential. Herein, two concentrations of catechol solution (2 mM and 20 mM) were successively added into the acetate buffer solution. It can be clearly seen that once the 100 µM of catechol was added, the response current increased instantly, the time for the current value reaching 95% of the next maximum response current value was only 3 s, indicating a fast response of biosensor, which may be attributed to the easy diffusion of catechol in the Lac-CXZnO-Nafion composite film. Figure 6B shows the calibration curve of response currents *vs.* catechol concentrations. The current values increased linearly with the ascent of catechol concentration. The linear range was from 6.91 µM to 453 µM with a correlation coefficient (*R*^2^) of 0.983 (*n* = 13). The sensitivity was 31.2 µA/mM and the detection limit was estimated to be 2.17 µM at a signal-to-noise of 3 (S/N = 3). Table 1 compares the biosensing performance of different laccase based biosensors. Our biosensor showed satisfactory detection results toward catechol with low detection limit, high sensitivity and wide linear range.

**Figure 7:**
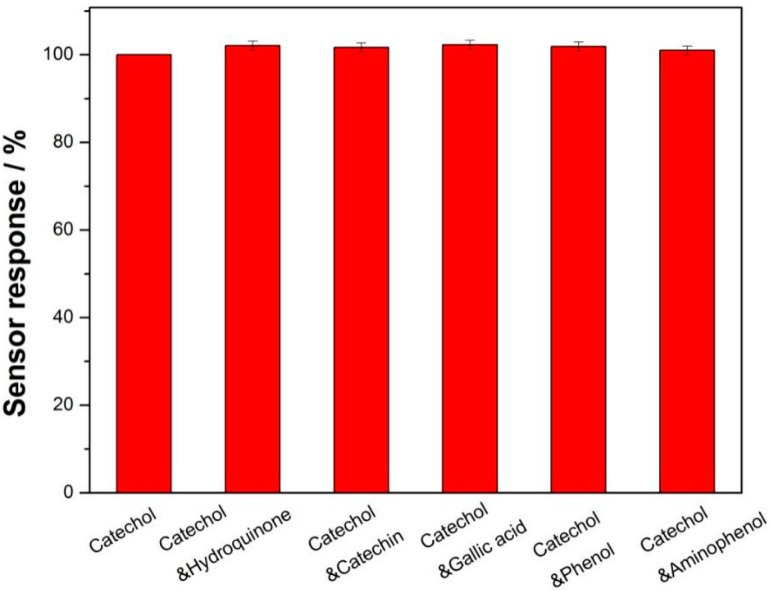
Selectivity of the GCE/Lac-CXZnO-Nafion in pH = 5.0 acetate buffer solution containing different phenolic compounds.

The fabrication reproducibility of the GCE/Lac-CXZnO-Nafion was investigated by successive detection of 100 µM catechol by six modified electrodes prepared in the same way. The relative standard deviation (RSD) was 3.2%, implying the acceptable reproducibility of the GCE/Lac-CXZnO-Nafion. The RSD of the GCE/Lac-CXZnO-Nafion for 20 times of successive detection of 100 µM catechol was 1.7%, indicating excellent repeatability of the GCE/Lac-CXZnO-Nafion. The selectivity experimental result is shown in Figure 7. The current response of the GCE/Lac-CXZnO-Nafion for 100 µM catechol solution and 100 µM catechol solution containing 100 µM interferents (hydroquinone, catechin, gallic acid, phenol, and aminophenol) was measured, respectively. Apparently, these interferents almost produced no effects on the current response of the biosensor, indicating the good selectivity of the GCE/Lac-CXZnO-Nafion.

**Figure 8:**
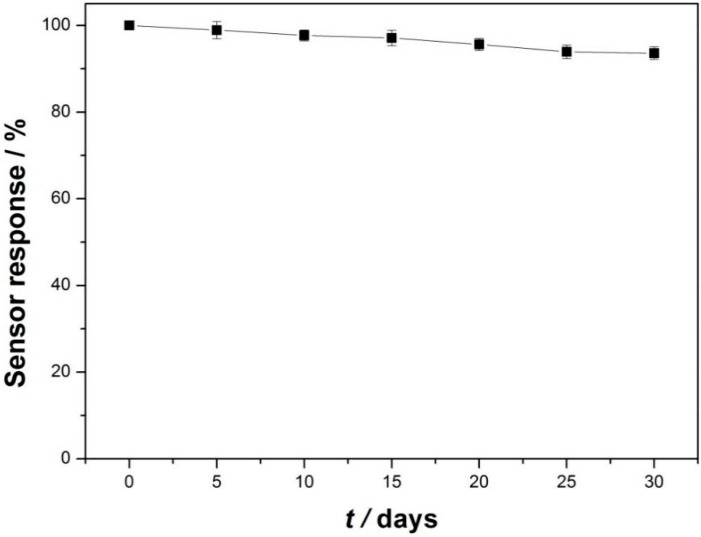
Storage stability of the GCE/Lac-CXZnO-Nafion in pH = 5.0 acetate buffer solution at 4 °C.

It can be seen from Figure 8 that the storage stability of the GCE/Lac-CXZnO-Nafion in pH = 5.0 acetate buffer solution at 4 °C was satisfactory. Furthermore, over one month of storage, the response current value could retain 93.6% of the original value, suggesting the excellent storage stability of the GCE/Lac-CXZnO-Nafion.

### 3.4. Real Sample Analysis

To test the practical application of the biosensor, we conducted recovery experiments using real lake water from Taihu Lake, Wuxi, China. An amount of 100 µM of catechol was added into the lake water samples, which was named C_added_. The determined content was denominated C_found_ and the result is shown in Table 2. It is seen that the recovery was very close to 100%, and the RSD was only 2.52%. This real sample analysis test demonstrated that the as-prepared biosensor can be successfully applied for the trace detection of catechol in a lake water environment.

## 4. Conclusions

In summary, a sol-gel condensation polymerization of formaldehyde and resorcinol solution containing zinc salt was used to prepare the precursors of CXZnO composites. The precursors were further dried and carbonized to obtain the CXZnO composites. A novel biosensor was fabricated by modifying a mixture containing CXZnO composites, Lac, and Nafion on the electrode surface. The as-prepared biosensor showed very good biological electrocatalysis towards catechol with high sensitivity, low detection limit, and wide linear range. Besides, the sensor was successfully used in trace detection of catechol existing in the real lake water environment. This novel composite further expands the application of carbon xerogel materials in the field of biosensing, and paves the way to develop highly sensitive phenolic biosensors.

## Figures and Tables

**Table 1:** Biosensing performance comparison of different laccase based biosensors toward catechol ^a^.

Electrodes	Detection Limit (µM)	Linear Range (µM)	Sensitivity (µA/mM)	Ref.
MB-MCM-41/PVA/lac	0.331	4–87.98	–	[19]
GCE/MCN/Tyr	0.01	0.05–12.5	–	[20]
Lac/AP- rGOs/Chit/GCE	7	15–700	15.79	[21]
Lac-FSM7.0-GC	2	2–100	–	[22]
Cu-OMC/Lac/CS/Au	0.67	0.67–13.8	104	[23]
GCE/Lac-CXZnO-Nafion	2.17	6.91–453	31.2	This work

^a^ The dashes in the table represent values that were not reported in the references.

**Table 2:** Determination of catechol content in real water samples (*n* = 5).

Sample	C_added_ (µM)	C_found_ (µM)	Recovery (%)	RSD (%)
a	100.00	103.26	103.26	2.52
–	100.00	98.93	98.93	–
–	100.00	97.31	97.31	–
–	100.00	102.47	102.47	–
–	100.00	101.95	101.95	–

a: Taihu Lake water.
